# Supplementary material for: Differentiation Capacity of Porcine Skeletal Muscle-Derived Stem Cells as Intermediate Species between Mice and Humans
Source: Int J Mol Sci. 2023 Jun 7;24(12):9862. doi: 10.3390/ijms24129862 (PMC10297882; doi:10.3390/ijms24129862)
Supplement: Supplementary file 1 [file ijms-24-09862-s001.zip › ijms-2418318-supplementary.pdf]

Table S1. Pig PCR primers.

	Gene Name		Product Size	
1	MyoD	pg_myod_F	152	TCCGACGGCATGATGGATTATAG
		pg_myod_R		TCCACGATGCTGGACAGACAG
2	Myf5	pg_myf5_F	218	GCATGCCTGAATGCAACAGC
		pg_myf5_R		AGAATCGGTGCTGGCAACTG
3	Pax3	pg_Pax3_F	158	GCACCAGGCATGGATTTTCC
		pg_Pax3_R		ATAGTCGGTCTGCGGCTGATG
4	Pax7	pg_Pax7_F	116	AGCTTCTCCAGCTACTCCGACAG
		pg_Pax7_R		GCACTGGGGTTGCTCAGAATG
5	M-cad	pg_Mcad_F	119	AGCGTCATCCTGCTGCTCTTG
		pg_Mcad_R		AGGATGTTGTCCCGGAGATCG
6	Myogenin	pg_myog_F	231	AGCGCCATCCAGTACATCGAG
		pg_myog_R		GGAGTGCAGATTGTGGGCATC
7	Skeletal muscle actin	pg_a_skel_act_2F	470	CCACAACGTGCCCATCTATGA
		pg_a_skel_act_2R		TCTCCTTCTGCATGCGGTCA
8	NCAM1	pg_NCAM1_F	158	ATCTCATGGTTCCGCGATGG
		pg_NCAM1_R		TCCTGTCCAATGCGGTTCAC
9	Cacnb1	pg_Cacnb1_F	211	TCGAGCGAATCTTCGAGCTG
		pg_Cacnb1_R		TTCTCCGAGGCCGCTATTTG
10	NG2	pg_NG2_F	109	TCAACGGACGGGTATTGCTG
		pg_NG2_R		TCCAGGGCTCCTTTGTGTGAG
11	Pmp22	pg_Pmp22_F	197	TGGCAAAACTGCACCACCTC
		pg_Pmp22_R		ATCTGGAAGACCCCGGTGATG
12	p75	pg_p75_F	116	ACCGACAACCTCATCCCTGTC
		pg_p75_R		TTGGCTCCTTGCTTGTTCTGC
13	Sox10	pg_Sox10_F	157	TCAGACGGAAACCCTGAGCAC
		pg_Sox10_R		CGTTCCCGAAGTCGATGTGAG
14	BDNF	pg_BDNF_F	222	CCAGAAAGTTCGGCCCAATG
		pg_BDNF_R		ACCCACTCGCTAATGCTGTCG
15	GDNF	pg_GDNF_F	127	TGACTTGGGTTTGGGCTACG
		pg_GDNF_R		CACCAGCCGTCTGTTTTTGG
16	Ninjurin	pg_Ninjurin_F	123	CCAGCTTCGCCTTCTTCATCC
		pg_Ninjurin_R		CATGCTTGGCAGGGTTGTTG
17	a-SMA	pg_aSMA_F	138	CTTCGTGTTGCCCCAGAAGA
		pg_aSMA_R		CACCGCCTGAATAGCCACAT
18	Smoothelin	pg_smoothelin_F	175	TGAAGACCACGTTCACCATCG
		pg_smoothelin_R		GGGCTGCTGATATGGGTGATG
19	VE-cad	pg_VEcad_F	176	GACAGCAACTTCACCCTCACG
		pg_VEcad_R		TTGCACTTGCACACGGTCAC
20	VEGF	pg_VEGFA_F	193	TACTGCCGTCCAATCGAGACC
		pg_VEGFA_R		GGCCTTGGTGAGGTTTGATCC
21	CD34	pg_CD34_F	238	TCCTTGCTCCTTGCCCAATC
		pg_CD34_R		AGCCAGTGATGCCCAAGACAG
22	GAPDH	pg_GAPDH_F	260	TCGGAGTGAACGGATTTGG
		pg_GAPDH_R		CGTGGGTGGAATCATACTGG
